# Persistence of plasmid and *tet*(X4) in an *Escherichia coli* isolate coharboring *bla*_NDM-5_ and *mcr-1* after acquiring an IncFII *tet*(X4)-positive plasmid

**DOI:** 10.3389/fmicb.2022.1010387

**Published:** 2022-10-19

**Authors:** Xia Xiao, Ziyi Liu, Xiaojun Chen, Kai Peng, Ruichao Li, Yuan Liu, Zhiqiang Wang

**Affiliations:** ^1^College of Veterinary Medicine, Yangzhou University, Yangzhou, Jiangsu, China; ^2^Jiangsu Co-Innovation Center for Prevention and Control of Important Animal Infectious Diseases and Zoonoses, Yangzhou, Jiangsu, China; ^3^Institute of Comparative Medicine, Yangzhou University, Yangzhou, Jiangsu, China

**Keywords:** *bla*_NDM-5_, *mcr-1*, *tet*(X4)-bearing plasmid, plasmid stability, *tet*(X4) stability

## Abstract

The prevalence of plasmid-mediated tigecycline resistance gene *tet*(X4) is presenting an increasing trend. Once *tet*(X4)-bearing plasmids are captured by multidrug-resistant bacteria, such as *bla*_NDM_ and *mcr*-coharboring bacteria, it will promote bacteria to develop an ultra-broad resistance spectrum, limiting clinical treatment options. However, little is known about the destiny of such bacteria or how they will evolve in the future. Herein, we constructed a multidrug-resistant bacteria coharboring *tet*(X4), *bla*_NDM-5_, and *mcr-1* by introducing a *tet*(X4)-bearing plasmid into a *bla*_NDM-5_ and *mcr-1* positive *E. coli* strain. Subsequently, the stability of *tet*(X4) and the plasmid was measured after being evolved under tigecycline or antibiotic-free circumstance. Interestingly, we observed both *tet*(X4)-bearing plasmids in tigecycline treated strains and non-tigecycline treated strains were stable, which might be jointly affected by the increased conjugation frequency and the structural alterations of the *tet*(X4)-positive plasmid. However, the stability of *tet*(X4) gene showed different scenarios in the two types of evolved strains. The *tet*(X4) gene in non-tigecycline treated strains was stable whereas the *tet*(X4) gene was discarded rapidly in tigecycline treated strains. Accordingly, we found the expression levels of *tet*(X4) gene in tigecycline-treated strains were several times higher than in non-tigecycline treated strains and ancestral strains, which might in turn impose a stronger burden on the host bacteria. SNPs analysis revealed that a myriad of mutations occurred in genes involving in conjugation transfer, and the missense mutation of *marR* gene in chromosome of tigecycline treated strains might account for the completely different stability of *tet*(X4)-bearing plasmid and *tet*(X4) gene. Collectively, these findings shed a light on the possibility of the emergence of multidrug resistant bacteria due to the transmission of *tet*(X4)-bearing plasmid, and highlighted that the antibiotic residues may be critical to the development of such bacteria.

## Introduction

Currently, the emergence of plasmid-mediated tigecycline high-level resistance gene *tet*(X) and its variants complicates the antimicrobial resistance (AMR) issue, which means that the use of several critical antibiotics, such as carbapenems, polymyxins, and tigecycline in clinical settings, has been restricted by their corresponding epidemic AMR genes. Generally, AMR genes conferring resistance to carbapenems, polymyxins, and tigecycline simultaneously present in a single pathogen is unusual. Now, however, the situation has begun to deteriorate. The isolate coharboring *tet*(X4) and *bla*_NDM_ has been described in *E. coli* (Sun et al., 2021), *Acinetobacter* spp. (Cui et al., 2020), *Proteus cibarius* (Li Y. et al., 2020), *K. aerogenes* (Hirabayashi et al., 2021), and *E. cloacae* (Li et al., 2022). Furthermore, the coexistence of *tet*(X4), *mcr-1*, and *bla*_NDM-5_ in a single *E. coli* isolate has been identified as well (Lu et al., 2022), highlighting these bacteria are constantly evolving. Nevertheless, there is a lack of relevant studies that systematically evaluate the possibility, evolutionary trend, and destiny of *bla*_NDM_, *mcr-1*, and *tet*(X4) in a single strain.

Plasmids play an indispensable role in spreading AMR genes and driving its rapid development (Rozwandowicz et al., 2018). Despite plasmids confer benefits to host, they also pose an extra burden (fitness cost) on the host (San Millan and MacLean, 2017). Fitness cost is one of the most important factors that affects plasmid persistence (Carroll and Wong, 2018). However, plasmids or host could alleviate the fitness cost by compensatory evolution under different circumstances, thereby improving the stability of plasmids. For example, large multidrug resistance plasmids could improve their stability through deleting the expensive region to offset fitness cost (Porse et al., 2016; Dorado-Morales et al., 2021). Single nucleotide substitution in the IncP-1 plasmid replication gene *trfA* increased plasmid stability and enhanced adaptation to new hosts (Sota et al., 2010). Mutations in the *uvrD* gene encoding helicase and β-subunit *rpoB* gene encoding RNA polymerase reduced host-plasmid replication protein interactions, which promoted the plasmid stability (Loftie-Eaton et al., 2017). Moreover, many studies demonstrated that the plasmid-host coevolution enhanced their mutual adaption, resulting in the plasmid persistence (Millan et al., 2014; Yano et al., 2016; Hall James et al., 2017). Given that *tet*(X4) gene is a newly identified AMR gene in recent years, and its transmission is predominantly mediated by plasmids. Hence, it is of great significance to investigate the fitness cost and compensatory evolution induced by *tet*(X4)-positive plasmids in host bacteria. Our previous study revealed that the *tet*(X4)-harboring IncFII plasmid showed the lowest fitness cost in TOP10 compared with other type of plasmids carrying *tet*(X4) (Tang et al., 2022). Besides, *E. coli* N31 carrying *bla*_NDM-5_ and *mcr-1* was isolated from swine feces in our early research project (data not published). Based on this background, we transferred the conjugative *tet*(X4)-positive IncFII plasmid pF65-*tet*(X4) into *E. coli* N31 to evaluate the possibility and the evolution pattern of the superbug carrying these three important AMR genes, and further verified whether positive selection would favor the stability of the plasmid and *tet*(X4) gene.

## Materials and methods

### Bacterial strains, plasmids, and media

*E. coli* N31 and *E. coli* F65 used in this study were isolated from pig farm and porcine slaughterhouse, respectively. The *tet*(X4)-positive IncFII plasmid pF65-*tet*(X4) (The original article was labeled as pRF65-1_113k_*tet*X) carried by F65 was regarded as the focal plasmid (Li R. et al., 2020), whereas *E. coli* N31 with *mcr-1* on chromosome, *bla*_NDM-5_ on IncX3 plasmid served as the recipient strain. The transconjugant CN31-pF65-*tet*(X4) was obtained through conjugation assay. All strains and plasmids used in this study were listed in Table 1. All strains were grown in the LB medium at 37°C.

**Table 1:** Basic information of bacteria and plasmids used in this study.

Name	Description	Source/Reference
Strains		
*E. coli* N31	As a recipient strain, in which *mcr-1* located on chromosome, whereas *bla*_NDM-5_ located on IncX3 plasmid. The remaining plasmids replicon type were IncFIB (K), IncFIB (AP001918), ColE10, and IncFIA (HI1).	Pig farm
*E. coli* F65	As a donor strain, in which *tet*(X4) located on IncFII plasmid. The remaining plasmid replicon type were IncFIB (K), IncFIA (HI1), and IncX1.	Porcine slaughterhouse (Li R. et al., 2020)
CN31-pF65-*tet*(X4)	N31 corresponding transconjugant carrying pF65-*tet*(X4).	In this study
CN31-F65-*tet*(X4)-E100^-T^-1/−2/−3/−4/−5	Five evolved clones selected from 100th generation populations evolved without tigecycline.	In this study
CN31-F65-*tet*(X4)-E100^+T^-1/−2/−3/−4/−5	Five evolved clones selected from 100th generation populations evolved with tigecycline (2 mg/l).	In this study
Plasmids		
pCN31-F65-*tet*(X4)	The ancestral plasmid from F65.	In this study
pCN31-F65-*tet*(X4)-E100^-T^ − 1/−2/−3/−4/−5	Five plasmids from the evolved strains without tigecycline.	In this study
pCN31-F65-*tet*(X4)-E100^+T^ − 1/−2/−3/−4/−5	Five plasmids from the evolved strains with tigecycline.	In this study

### Evolution experiment

Serial passage was conducted using the transconjugant CN31-pF65-*tet*(X4). Briefly, the ancestral strain was propagated in 5 ml LB broth with or without tigecycline (2 mg/l) at 37°C. Every 12 h, 5 μl of each culture was transferred into 5 ml fresh corresponding LB broth. Each passaging was defined as one generation evolution. The evolution experiment lasted 50 days to yield 100 generations. At the end of evolution, the populations passaged with and without tigecycline were plated on Maconkey agar medium containing tigecycline and meropenem (2 mg/l) to randomly select five evolved clones containing *tet*(X4) gene. These selected strains were named as CN31-pF65-*tet*(X4)-E100^+T-1/−2/−3/−4/−5^ and CN31-pF65-*tet*(X4)-E100^-T-1/−2/−3/−4/−5^, respectively.

### Plasmid and *tet*(X4) loss frequency assays

To investigate the stability of the plasmid and *tet*(X4) gene, the selected evolved strains (CN31-pF65-*tet*(X4)-E100^+T-1/−2/−3/−4/−5^, CN31-pF65-*tet*(X4)-E100^-T-1/−2/−3/−4/−5^) were further passaged in antibiotic-free LB broth using the same strategy described in the evolution experiment. After 60 generations, the cultures were diluted and streaked on antibiotic-free LB plates and incubated for 12 h at 37°C. The strains containing plasmid- or *tet*(X4)-containing were determined by randomly selecting 50 single colonies to perform PCR with specific primers. The primers for IncFII were forward (FV)5′-GGCGAAATCAAAACGGGAGG and reverse (RV) 5′-CGATGCATGTGATGATGGGC. The primers for *tet*(X4) were forward (FV)5′-TGAACCTGGTAAGAAGAAGTG and reverse (RV) 5′-CCGACAATATCAAGGCATCCA. Plasmid and *tet*(X4) loss rates were determined by dividing the clones without a specific primer band by the 50 clones.

### MIC determination

The MICs of tigecycline against the ancestral strain (CN31-pF65-*tet*(X4)) and evolved strains (CN31-pF65-*tet*(X4)-E100^+T-1/−2/−3/−4/−5^, CN31-pF65-*tet*(X4)-E100^-T-1/−2/−3/−4/−5^) were determined using the broth microdilution method according to the Clinical and Laboratory Standards Institute (CLSI) guidelines (Zatyka et al., 1997) and interpreted following the criteria of European Committee on Antimicrobial Susceptibility Testing (Version 11.0).^1^ *E. coli* ATCC25922 was used as the quality control strain.

### Time killing curve

The ancestral strain (CN31-pF65-*tet*(X4)) and evolved strains (CN31-pF65-*tet*(X4)-E100^+T-1/−2/−3/−4/−5^ and CN31-pF65-*tet*(X4)-E100^-T-1/−2/−3/−4/−5^) were added with tigecycline at the concentration of 0, 4, 8, 16, 24, 32 mg/l and cultured at 37°C with shaking at 200 rpm. An aliquot of 100 μl mixtures were taken out after being cultured for 4, 12, 24 h, and then were tenfold serially diluted and plated on LB plates to calculate the colony-forming units (CFUs) after incubation at 37°C for 24 h.

### Plasmid conjugation assay

To investigate the transferability of ancestral and evolved strains, conjugation assay was performed as described previously (Li Y. et al., 2020). Briefly, the ancestral strain (CN31-pF65-*tet*(X4)) and evolved strains (CN31-pF65-*tet*(X4)-E100^+T-1/−2/−3/−4/−5^ and CN31-pF65-*tet*(X4)-E100^-T-1/−2/−3/−4/−5^) were served as the donor strains and *E. coli* C600 (resistant to rifampin) as the recipient strains. Cultures of donor and recipient strain with a density of 0.5 McFarland were mixed at a ratio of 1:4, respectively. Subsequently, 0.1 ml of the mixed cultures was applied onto a sterile filtration membrane. The membrane was cultured in LB agar plates at 37°C for 12 h. Following the bacteria on the membrane was collected and diluted with sterile saline, and then plated on the LB plates containing 300 mg/l rifampin or 2 mg/l tigecycline and 300 mg/l rifampin. The number of transconjugants carrying *tet*(X4) were calculated after incubation at 37°C for 24 h. The presence of *tet*(X4) in transconjugants was confirmed by PCR. Conjugation frequencies were calculated by the number of transconjugants per recipient cell.

### Gene expression analysis

The RNA isolater Total RNA extraction Reagent (Vazyme, Nanjing, China) was used to extract total RNA of the ancestral strain (CN31-pF65-*tet*(X4)) and evolved strains (CN31-pF65-*tet*(X4)-E100^+T-1/−2/−3/−4/−5^ and CN31-pF65-*tet*(X4)-E100^-T-1/−2/−3/−4/−5^). The extracted RNA was reverse transcribed into cDNA using a HiScript III RT Supermix for qPCR (+g DNA wiper) (Vazyme. Nanjing, China). Real-time quantitative PCR (qPCR) was used to quantify gene expression. The expression levels of *tet*(X4) gene, conjugative transfer regulatory genes *traJ*, *traY*, *traM*, and *traI*, conjugative transfer protein genes *traD*, *trbB*, *trbI*, and *vird2*, conjugative pilus assembly genes *traB*, *traC*, *traG*, *traE*, *traW*, *traP*, *traH*, *traV*, and *traX* were determined using ChamQ SYBR color qPCR master Mix (Vazyme, Nanjing, China) in triplicate. 16S rRNA was used as the internal control. Primer sequences are listed in Supplementary Table S1. The qPCR primers were synthesized by Tsingke Biotech Co., Ltd. (Nanjing, China), and qPCR was performed using a LineGene 9600 Plus real time PCR detection system (Bioer, Hangzhou, China).

### DNA sequencing and bioinformatics analysis

Genomic DNA of the ancestral strain (CN31-pF65-*tet*(X4)) and evolved strains (CN31-pF65-*tet*(X4)-E100^+T-1/−4/−5^ and CN31-pF65-*tet*(X4)-E100^-T-1/−2/−3^) were extracted using the TIANamp bacterial DNA kit (TianGen, Beijing, China) and subjected to short-read sequencing (2 × 150 bp) with the Illumina HiSeq 2500 platform. Short-read Illumina raw sequences of ancestral and evolved strains were separately assembled using SPAdes (Bankevich et al., 2012), and contigs less than 500 bp were discarded. SNP analysis was performed using Snippy (4.0.2) against the genome sequences of the ancestral strain.^2^

The plasmids of the ancestral strain and selected evolved strains CN31-pF65-*tet*(X4)-E100^+T-1^ and CN31-pF65-*tet*(X4)-E100^-T-2^ were extracted using Qiagen Plasmid Midi-Kit (Qiagen, Hilden, Germany). Subsequently, the plasmids were sequenced with the Oxford Nanopore Technologies MinION long-read platform. The Nanopore long-read MinION sequences of plasmids were subjected to *de novo* assembly with the Flye tool (Kolmogorov et al., 2019). Easyfig was used to describe the structural diversity of the ancestral and the evolved plasmids (Sullivan et al., 2011).

### Statistical analyses

GraphPad Prism 8.3.2 was used for data analyses. Data are expressed as mean ± standard deviation. Significant differences were assessed using a two-way analysis and *t* test, with *p* < 0.05 considered as statistically significant.

## Results

### The IncFII *tet*(X4)-positive plasmid imposes fitness cost in *Escherichia coli* N31

**Figure 1:**
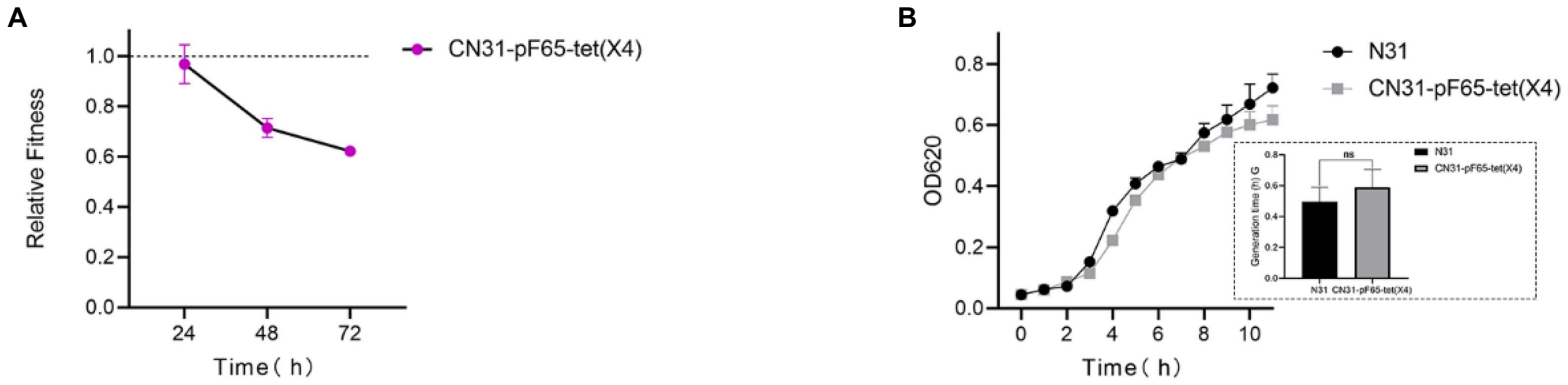
*tet*(X4)-positive plasmid imposes fitness cost on *E. coli* N31. **(A)** Relative fitness of *tet*(X4)-positive plasmid-carrying strains versus isogenic plasmid-free strains *in vitro*. **(B)** Comparison of growth curve and generation time (lower right corner) between *tet*(X4)-positive plasmid-carrying and plasmid-free strains.

To investigate the fitness cost of the IncFII *tet*(X4)-positive plasmid in wild-type multidrug-resistant *E. coli* N31 carrying *bla*_NDM-5_ and *mcr-1*, the plasmid was transferred into strain N31 by conjugation. The transconjugant was resistance to meropenem and colistin with the MICs value of 64 and 2 mg/l, respectively. Pairwise competitions results showed that the relative fitness of transconjugant was less than 1, suggesting the introduction of the plasmid weakened the competitiveness of N31 (Figure 1A). In terms of bacterial growth, there was no significant difference of the bacteria growth between the transconjugant and N31, yet the generation time of N31 was shorter than that of the transconjugant (Figure 1B). These findings indicated that the *tet*(X4)-positive plasmid incurred the fitness cost in N31. To get further insights into the stability of the IncFII *tet*(X4)-positive plasmid in N31, the transconjugant was passaged for 100 generations with or without tigecycline. We randomly selected five *tet*(X4)-positive single clones from the 100th generation population passaged with and without tigecycline, hereafter named CN31-pF65-*tet*(X4)-100^+T-1/−2/−3/−4/−5^ and CN31-pF65-*tet*(X4)-100^-T-1/−2/−3/−4/−5^, respectively for further explorations.

### Plasmid and *tet*(X4) gene stability of evolved strains in the presence or absence of tigecycline

**Figure 2:**
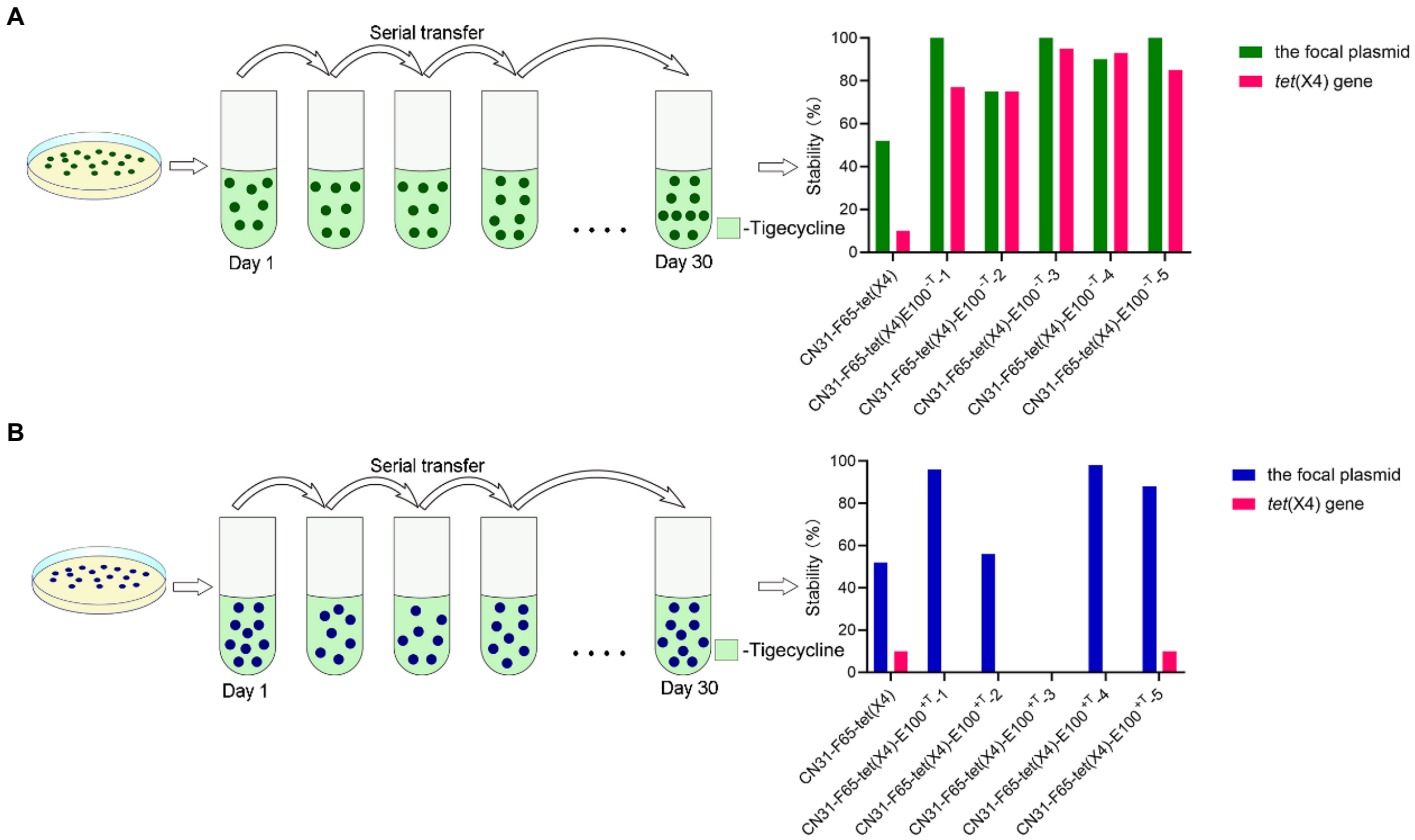
Stability of the focal plasmid and *tet*(X4) gene. **(A)** Stability of the focal plasmid and *tet*(X4) gene in five selected strains evolved in antibiotic-free broth and the ancestral strain at the 30^th^ day. **(B)** Stability of the focal plasmid and *tet*(X4) gene in five selected strains evolved in tigecycline containing broth and the ancestral strain at the 30^th^ day.

The evolved strains in the presence or absence of tigecycline were subjected to serial passage in antibiotic-free LB broth for 30 days. PCR targeting the plasmids and *tet*(X4) genes revealed that both the plasmid and *tet*(X4) gene in CN31-pF65-*tet*(X4)-100^-T^ were stable (the retention rate for the plasmid was 100, 75, 100, 90, and 100%, and *tet*(X4) gene was 77, 75, 95, 93, and 85% in CN31-pF65- *tet*(X4)-100^-T-1/−2/−3/−4/−5^, respectively), while the plasmid in partial CN31-pF65-*tet*(X4)-100^+T^ (3/5) was stable but the *tet*(X4) gene was readily lost and independent of the plasmid (the retention rate for the plasmid was 96, 56, 0, 98, and 88%, and *tet*(X4) gene was 0, 0, 0, 0, and 10% in CN31-pF65- *tet*(X4)-100^+T-1/−2/−3/−4/−5^, respectively: Figure 2). Notably, we did not observe the loss of native plasmids in N31 during passaging, indicating that these plasmids and host bacteria have perfectly adapted to each other.

### The underlying mechanisms resulting in the low retention rate of *tet*(X4) in strains evolved with tigecycline

**Figure 3:**
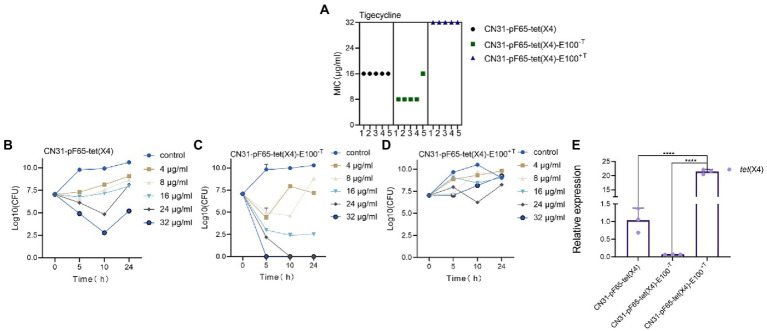
Evolved strains with different resistance levels to tigecycline under selective and non-selective pressure. **(A)** Comparison of MIC of tigecycline between ancestral and evolved strains. **(B–D)** Time-kill curve of tigecycline for the ancestral strain **(B)**, evolved strains without tigecycline **(C)**, and evolved strains with tigecycline **(D)**. **(E)** Comparison of the expression levels of *tet*(X4) gene between ancestral and evolved strains.

In order to evaluate the tigecycline resistance levels between the two types of evolved strains, we performed antimicrobial susceptibility testing for tigecycline. The MIC value of tigecycline was 16 μg/ml for ancestral strains (5/5), 8 μg/ml for CN31-pF65-*tet*(X4)-100^-T^ (4/5), and 32 μg/ml for CN31-pF65-*tet*(X4)-100^+T^ (5/5) (Figure 3A). Additionally, the killing curve of tigecycline demonstrated that CN31-pF65-*tet*(X4)-100^-T^ was more susceptible to tigecycline with a 4–log_10_ reduction at tigecycline concentration of 16 μg/ml while tigecycline only exhibit a bacteriostatic effect even at concentration of 32 μg/ml against CN31-pF65-*tet*(X4)-100^+T^ (Figures 3B–D). These results indicated that the instability of *tet*(X4) in evolved strains under tigecycline pressure may attribute to its high expression level. To verify the hypothesis, we measured the mRNA expression of *tet*(X4). The *tet*(X4) expression levels were 20-fold higher in CN31-pF65-*tet*(X4)-100^+T^ compared to the ancestral strain, yet the *tet*(X4) expression was significantly suppressed in CN31-pF65-*tet*(X4)-100^-T^ (Figure 3E), suggesting the evolution under the tigecycline pressure would improve the expression of *tet*(X4) gene which in turn enhanced the resistance level to tigecycline. Accordingly, the high expression of *tet*(X4) gene supposed to impose high burden on the cell and led to low retention rate of *tet*(X4). However, the scenario was totally different in the strain evolved without tigecycline.

### Changes in conjugation frequency of the focal plasmid during evolution

**Figure 4:**
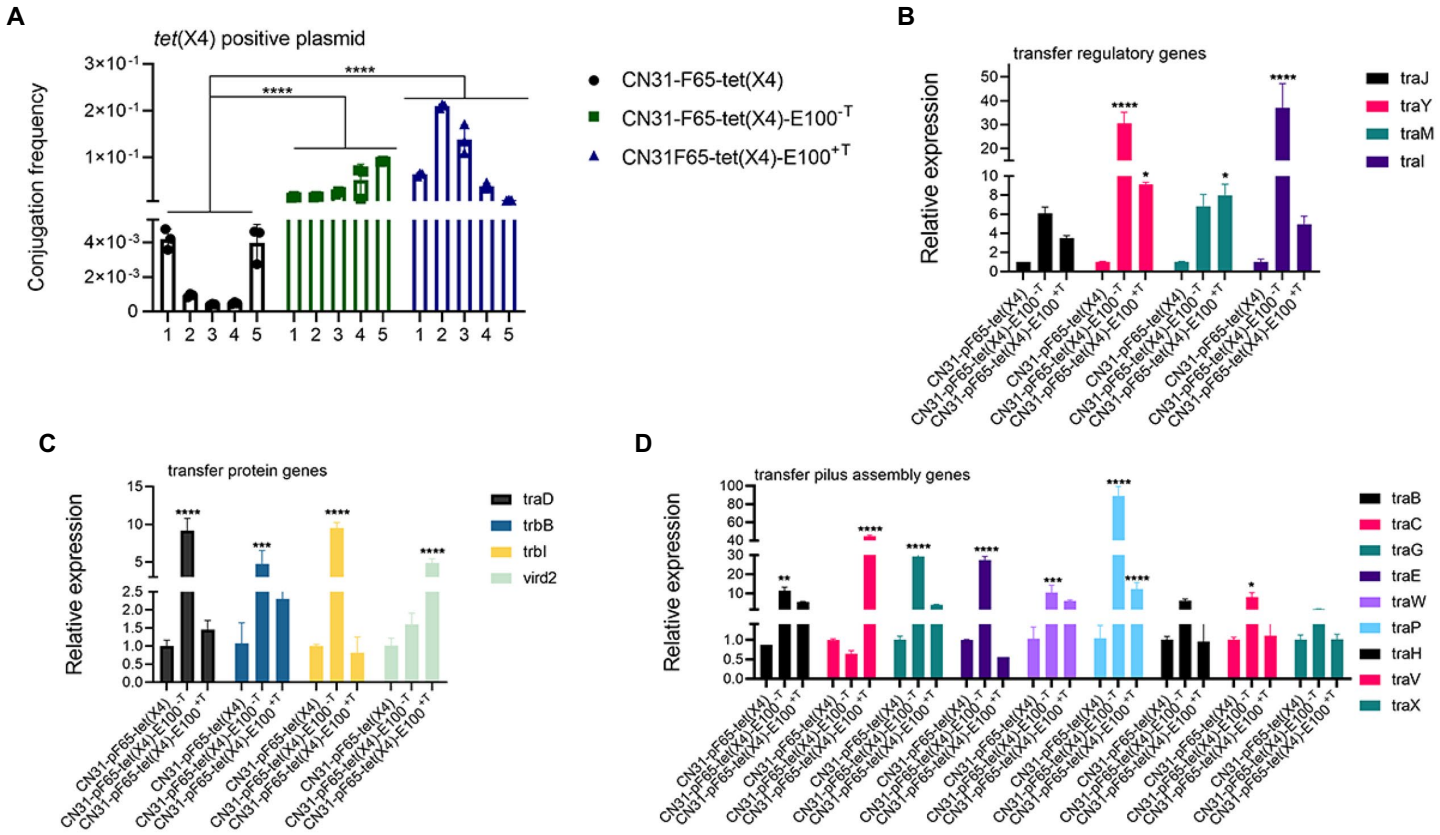
Improved conjugation frequencies observed in the evolved strains. **(A)** Comparison of *tet*(X4)-positive plasmid conjugation frequency between ancestral and evolved strains. **(B)** Comparison of the expression levels of conjugative transfer regulatory-related genes between ancestral and evolved strains. **(C)** Comparison of the expression levels of conjugative transfer genes between ancestral and evolved strains. **(D)** Comparison of the expression levels of conjugative transfer pilus assembly genes between ancestral and evolved strains.

It was reported that the stability of plasmids could be improved through high conjugation rate (Carroll and Wong, 2018). Thus, the conjugation frequencies of evolved strains were examined to gain insight into plasmid evolution. Interestingly, the conjugation rate of *tet*(X4)-positive plasmid increased significantly in both tigecycline and non-tigecycline evolved strains (Figure 4A), as the conjugation frequency of the plasmid increased from 2 × 10^−3^ ± 0.001914 (average in the ancestral strains) to 3.93 × 10^−2^ ± 0.032495 (average in the evolved strains without tigecycline) and 9.13 × 10^−2^ ± 0.081851 (average in the evolved strains with tigecycline). Additionally, the conjugative transfer regulatory genes *traJ*, *traY*, *traM*, and *traI* were upregulated in both evolved strains, promoting transcription of downstream genes encoding conjugative-related genes such as pilus assembly genes, T4SS secretion system genes, and others (Figures 4B–D).

### Deletion of multidrug resistance regions promotes plasmid stability

**Figure 5:**
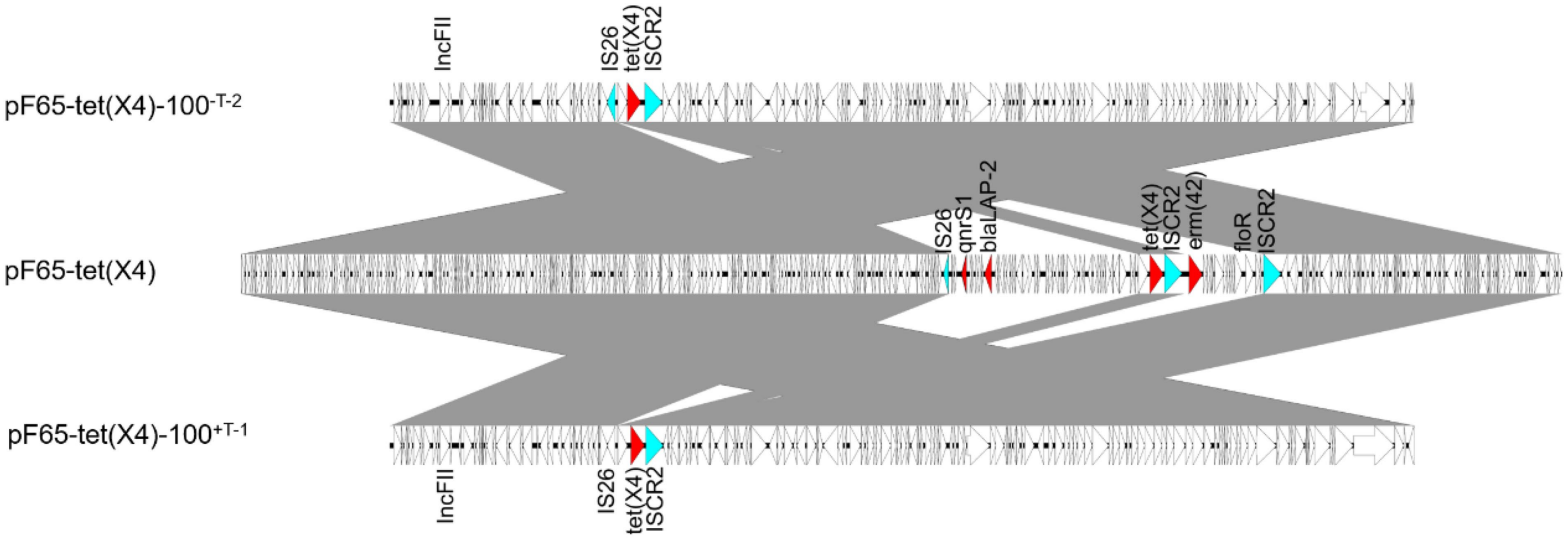
Linear comparison among pF65-*tet*(X4) and two evolved plasmids. Two segments were discarded during evolution, and the AMR genes and insertion sequences in deleted regions were marked in red and blue, respectively.

To further analyze the structural basis responsible for the enhancement of plasmid stability, we sequenced the evolved plasmid pF65-*tet*(X4)-100^-T-2^ without tigecycline and the evolved plasmid pF65-*tet*(X4)-100^+T-1^ with tigecycline. Compared with the ancestral plasmid, a consistent segment of approximately 22 kb was discarded in the two evolved plasmids, and no obvious structural differences were found between them, suggesting they experienced the same evolutionary trajectory, despite evolved in different circumstances. The deleted region contained two fragments, one of which carried *floR* and *erm*(42), whose loss was mediated by homologous recombination of IS*CR2*, whereas the deletion of the other fragment harboring *qnrS1* and *bla*_LAP-2_ may be attributed to the IS*26* activity (Figure 5).

In order to verify the stability of structural reorganized plasmids in other strains, we transformed the ancestral and evolved plasmids into the engineering strain TOP10. As predicted, the stability of evolved plasmids was significantly improved, which is much higher than that of the ancestral plasmid in TOP10. Moreover, the retention rates of *tet*(X4) gene were also improved in evolved plasmids (Supplementary Figure S1A), suggesting the stability of *tet*(X4) may also be influenced by genetic characteristics of the host bacteria. Furthermore, the conjugation frequencies of the evolved plasmid were increased in different extent compared to the ancestral plasmids in TOP10 (Supplementary Figure S1B). These results illustrated that such structural alterations may influence on the horizontal transfer and stability of the plasmid.

### SNP analysis revealed the mutations of chromosome and plasmid may co-drive the evolutionary process

**Figure 6:**
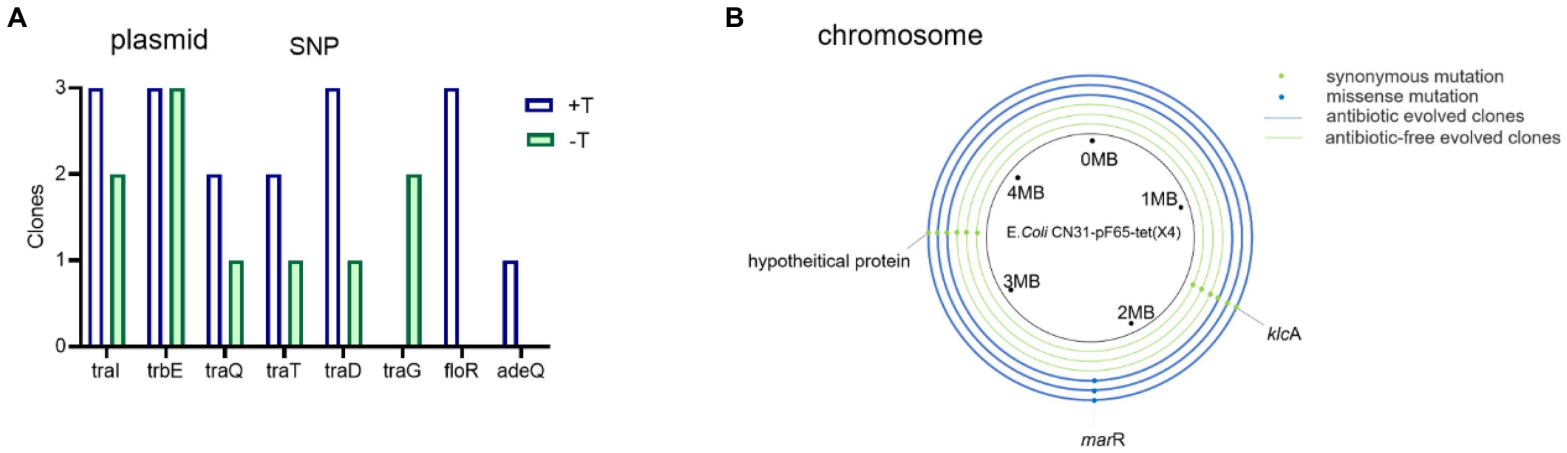
Genomic mutations associated with conjugative transfer-related genes and antibiotic resistance genes on plasmid and chromosome. **(A)** Mutations observed on plasmid of evolved clones. **(B)** Mutations observed on chromosome of evolved clones. Blue rings represent the antibiotic evolved clones and the green rings represent the antibiotic-free evolved clones. Dots represent the mutations.

The accumulations of genomic mutations were powerful evidences to reveal the mechanism of bacterial evolution, hence we sequenced the whole-genomes of the ancestral strain, three evolved bacteria in the absence of tigecycline, and three evolved bacteria in the presence of tigecycline. All SNPs occurring in coding sequences were listed in Supplementary Table S2. Strikingly, the single nucleotide polymorphisms (SNPs) in the plasmid were found in genes involving in the conjugative transfer and antibiotic resistance. Mutations in plasmid *tra* genes were observed, which are conjugative transfer-related genes that play an important role in conjugation transfer by encoding transfer proteins, transfer regulatory, and transfer surface exclusion protein genes. Other SNPs on the plasmid included the *adeQ* gene, which encodes adenine permease, and *floR* gene, conferring florfenicol resistance (Figure 6A). For the chromosome, a missense mutation of *marR* which encoded a multiple antibiotic resistance suppressor was identified in all strains evolved with tigecycline (Figure 6B). Functional prediction *via* PROVEAN suggested the altered residue may has deleterious effect on protein functions (Choi et al., 2012). Collectively, the mutations observed in the chromosome and plasmid of evolved strains indicated that the shift of phenotype in evolved strains may due to the plasmid-host coevolution.

## Discussion

The emergence and prevalence of multidrug-resistant (MDR) bacteria threaten the efficacy of antibiotics. Conjugative plasmids, which could transfer antimicrobial resistance (AMR) genes among bacteria, played a major role in MDR bacteria formation (Frost et al., 2005; Soucy et al., 2015; Mccollister et al., 2016). Nevertheless, the acquisition of exogenous plasmids or AMR genes may cause fitness cost to host, and expect to go extinct. Thus, the persistence of plasmids and AMR genes in host cell was critical for the formation and prevalence of MDR bacteria. It was reported that positive selection would favor the maintenance of plasmid (Stevenson et al., 2018). However, little was known about whether it would favor the stability AMR genes. The emergence and dissemination of *bla*_NDM_, *mcr*, and *tet*(X4), have seriously undermined the effectiveness of “the last resort antibiotics” in clinical settings (He et al., 2019a,b; Zhi et al., 2020; Mm et al., 2021; Sun et al., 2021a). To evaluate whether “superbugs” co-bearing *tet*(X4), *mcr-1*, and *bla*_NDM-5_ genes will prevalent in clinical settings, a *tet*(X4)-positive IncFII plasmid, a main prevalence type in China (Li et al., 2021), was introduced into a wide-type MDR *E.coli* N31 carrying *mcr-1* and *bla*_NDM-5_ genes.

Fitness cost and plasmid transfer rate are key parameters for predicting plasmid persistence. Initially, we observed the focal plasmid entailed a substantial burden in N31, and rapid plasmid extinction was found after 20th generation (data not shown). As fitness cost of plasmids could be alleviated either under non-selection or selection pressure (Porse et al., 2016; Wein et al., 2019), we passaged the strain in the absence or presence of tigecycline. Surprisingly, the stability of both plasmid and *tet*(X4) gene in strains evolved without tigecycline was significantly elevated, whereas the stability of the plasmid in three out of five strains evolved with tigecycline was elevated. However, *tet*(X4) gene harbored by these strains was discarded rapidly, which implied the presence of tigecycline may adversely affect the stability of *tet*(X4) gene. This interesting phenomenon may be collectively caused by the combination of boosted expression of *tet*(X4), increased conjugation frequency, structural alterations of the *tet*(X4)-positive plasmid, and mutations of chromosomal and plasmid genes in evolved strains under tigecycline.

First, we observed the high expression of *tet*(X4) gene in tigecycline treated strains, which may directly contribute to the instability of *tet*(X4). However, the expression level of *tet*(X4) in non-tigecycline treated strains was significantly suppressed. This sharp contrast implied that the high-level expression of *tet*(X4) may arise biosynthetic burden for host bacteria. Previous study has demonstrated the expression of *tet*(X6) exerted high fitness cost in *E. coli* as well (Jiang et al., 2021), suggesting the expression of *tet*(X) variants may be positively correlated with fitness cost. Second, an elevated conjugation frequency was observed in both evolved strains, and the expression of genes involving in the conjugative process was increased as well. It is generally accepted that conjugation is energy-intensive process and reduces the host bacterial fitness (Ilangovan et al., 2015), thus the relationship between bacteria and plasmid should evolve towards lower plasmid costs rather than the high conjugation rate (Hall James et al., 2017). However, the focal plasmid belongs to IncFII type, which represents one of the most prevalent plasmid types in the dissemination of AMR genes (Muthuirulandi Sethuvel et al., 2019). It has perfect conjugation transfer system, partitioning system, *psiB* gene encoding SOS inhibitor protein, and *ssb* encoding DNA-binding protein, which may allow the plasmid to maximize their transfer function (Al Mamun et al., 2021). In addition, it was proved that if the plasmid transfer rate was high enough, it was sufficient to compensate for the loss of plasmids due to plasmid segregation and growth disadvantage and favor the persistence of plasmid (Stewart and Levin, 1977; Dionisio et al., 2002). Third, sequencing of both evolved plasmids revealed that the elevated plasmid stability was also achieved by a large-scale sequence deletion event. Such evolution strategy occurring in plasmids, especially multidrug resistant plasmids, was common. For example, a 73 kb conjugative multidrug resistance plasmid could delete a 25 kb costly region to expand its host range and improve its stability (Porse et al., 2016). Moreover, it was reported that plasmid could compensate its fitness cost to the host through losing 12.8 kb multidrug resistance region (Dorado-Morales et al., 2021). This series of deletion events may be caused by the burden of these genes in transcription, translation and subsequent interactions between these proteins and cellular network, and the loss of these regions may promote the maximum survival of plasmids (Ilangovan et al., 2015; San Millan and MacLean, 2017). Finally, the SNPs of evolved strains showed consistency. Missense mutation in *marR* gene occurred in chromosome of all strains evolved in tigecycline. The *marR* gene is a member of multiple antibiotic resistance repressor (MarR) family, which acts as a regulator to modulate the multidrug resistance efflux pump (Barbosa and Levy, 2000; Lisa et al., 2020). As previously reported, *marR* regulates the AcrAB multidrug efflux pump through regulation of *marA*, enabling cellular resistance to many structurally unrelated antibiotics, including *tet*racycline, ampicillin, and chloramphenicol (Sato et al., 2018). Moreover, the inactivation mutations in the *marR* gene often associate with a significant fitness cost (Praski Alzrigat et al., 2021). Combining with our results, we hypothesize the mutation of *marR* may be another reason for the increased level of tigecycline resistance and improved plasmid stability in addition to the change of *tet*(X4) expression. Furthermore, plenty of SNPs occurred in conjugation-related genes in the *tet*(X4)-positive plasmids of all evolved strains, probably leading to the high expression of conjugation-related genes. However, further studies were needed to confirm this hypothesis.

In conclusion, this study found that antibiotic-free evolution can improve the stability of *tet*(X4) positive plasmid in *bla*_NDM-5_ and *mcr-1* positive *E. coli* by increasing their conjugation frequency and losing large costly fragments. However, the retention rate of *tet*(X4) was reduced through evolution under tigecycline, which was probably caused by the high expression of *tet*(X4). The results of the study provided a theoretical basis for further exploration of the formation of multidrug resistant bacteria and the dissemination of antibiotic resistance.

## Data availability statement

The datasets presented in this study can be found in online repositories. All available sequences have been submitted to the Figshare database (https://doi.org/10.6084/m9.figshare.20325096.v1).

## Author contributions

XX and ZW designed this study. XX, ZL, and XC did the experiment. KP, RL, and YL analyzed the data. XX and XC wrote the paper. ZL and ZW revised the paper. All authors contributed to the article and approved the submitted version.

## Funding

The project was supported by the Priority Academic Program Development of Jiangsu Higher Education Institutions (PAPD), China.

## Conflict of interest

The authors declare that the research was conducted in the absence of any commercial or financial relationships that could be construed as a potential conflict of interest.

## Publisher’s note

All claims expressed in this article are solely those of the authors and do not necessarily represent those of their affiliated organizations, or those of the publisher, the editors and the reviewers. Any product that may be evaluated in this article, or claim that may be made by its manufacturer, is not guaranteed or endorsed by the publisher.

## Footnotes

^1^ http://eucast.org/clinical_breakpoints/

^2^ http://github.com/tseemann/snippy
