# Supplementary material for: Persistence of plasmid and tet(X4) in an Escherichia coli isolate coharboring blaNDM-5 and mcr-1 after acquiring an IncFII tet(X4)-positive plasmid
Source: Front Microbiol. 2022 Oct 19;13:1010387. doi: 10.3389/fmicb.2022.1010387 (PMC9626518; doi:10.3389/fmicb.2022.1010387)
Supplement: Supplementary file 1 [file Data_Sheet_1.docx]

**Supplementary Data**

**Persistence of plasmid and *tet*(X4) in an *Escherichia coli* isolate coharboring *bla*_NDM-5_ and *mcr-1* after acquiring an IncFII *tet*(X4)-positive plasmid**

**Table S1. The primer sequences and product size.**

| Type | Genes | Primer (5’→3’) | Product(bp) |
| --- | --- | --- | --- |
| Resistance genes | *tet*(X4) | F:GGAACGCTACCAGCTACCTC | 151 |
|  |  | R:TACGATATGCGTCGTCTGGC |  |
|  | *bla*_NDM-5_ | F:CAACACAGCCTGACTTTCGC | 79 |
|  |  | R:AAAATACCTTGAGCGGGCCA |  |
| Entry exclusion genes | *traS* | F:GCCAGTATCTTCCTTACTCTCTCC | 91 |
|  |  | R:TACACAACCACTAATTCGCCT |  |
|  | *traT* | F:TCCGATTTAGCGATGCCTCC | 144 |
|  |  | R:CGCCAGCGAGACATACATCA |  |
| Transfer pilus assembly | *traB* | F:ACGCAA*C*GGGATGTTTTGTG | 85 |
| genes |  | R:CAACTGATATTGCGGGTGCG |  |
|  | *traC* | F:CTCTGCTGGCGTTACTGGTT | 159 |
|  |  | R:CAGACGGAGGCATCGCTAAA |  |
|  | *traG* | F:ATGACAGAGCAGAAGCGACC | 99 |
|  |  | R:TGGCGTGGTGACATTGATGA |  |
|  | *traE* | F:TTGCCGTGTCACAGAACAGT | 107 |
|  |  | R:ATGTGAGGCATCGACGGTTT |  |
|  | *traW* | F:TCGGACGCACGGAGAAATAC | 89 |
|  |  | R:AAACACCCGCCCTTCGTTAT |  |
|  | *traP* | F:TGTTCACCGCATTATGGGCA | 89 |
|  |  | R:CATTTTTCCGGGTCGCATCG |  |
|  | *traH* | F:AATTGTCGTTTCGCTGTGCC | 151 |
|  |  | R:CGGCGCATACGACATTTCAG |  |
|  | *traV* | F:GCACATCCGGAGCAGAAACT | 119 |
|  |  | R:GTTCTTAGCGGACGAGGAGG |  |
|  | *traX* | F:ATGACGGGACAGATTCAGCC | 99 |
|  |  | R:AACCGCATTCTGCATCTGGA |  |
| Transfer protein genes | *traD* | F:TGTTCAGCCAGATCGCCAAT | 135 |
|  |  | R:GCGTGGTACACCACCAGTAA |  |
|  | *trbB* | F:AATTGTCGTTTCGCTGTGCC | 151 |
|  |  | R:CGGCGCATACGACATTTCAG |  |
|  | *trbI* | F:AGAACAACGCATCCACTGGT | 96 |
|  |  | R:GCGTTCAGTCTGACAATGCC |  |
|  | *virD2* | F:TCCGCCGTGCTGGTTGTCGGA | 135 |
|  |  | R:TTTGGGACCGCAGTTGACTC |  |
| Regulatory genes | *traY* | F:GGTACACGTTCTGCAACAGG | 103 |
|  |  | R:ACGATCGACTTCTTCCGCTT |  |
|  | *traM* | F:TTGAGAAGCGTCGACAGGAAG | 91 |
|  |  | R:TGTACACGAAGCCCCAGTTC |  |
|  | *traI* | F:CAGCACGTCGATCCTGAAGT | 78 |
|  |  | R:ATATGCCCGGATGTCGAACC |  |
|  | *traJ* | F:TGGAAGCATACAGGAATGAGCA | 151 |
|  |  | R:TGACGAACATGAGCAGCATC |  |
| Reference gene | 16S | F:CCTACGGGAGGCAGCAG | 194 |
|  |  | R:ATTACCGCGGCTGCTGG |  |

**Table S2. SNPs identified in the coding sequence of evolved strains compared to the ancestral strain.**

| Strain | SNP | Nucleotide position | Product |
| --- | --- | --- | --- |
| CN31-pF65-tet(X4)-E100^-T-1^ | T→A | 21/261 | trbE |
|  | G→A | 15/282 | traQ |
|  | G→A | 1865/2823 | traG |
|  | G→A | 180/480 | klcA (chromosome) |
|  | A→G | 340/363 | hypothetical protein (chromosome) |
|  | T→C  G→A  A→C  T→G  T→C | 204/2190  221/2190  516/2190  1113/2190  2157/2190 | traD |
|  | T→A  C→T  A→G  T→C  A→G  T→C | 720/1878  1367/1878  1386/1878  1494/1878  1554/1878  1611/1878 | traI |
| CN31-pF65-tet(X4)-E100^-T-2^ | T→A | 21/261 | trbE |
|  | G→A  C→T | 696/777  762/777 | traT |
|  | G→A | 180/480 | klcA (chromosome) |
|  | A→G | 340/363 | Hypothetical protein (chromosome) |
| CN31-pF65-tet(X4)-E100^-T-3^ | T→A | 21/261 | trbE |
|  | G→A | 1865/2823 | traG |
|  | T→G  T→A | 375/1878  720/1878 | traI |
|  | G→A | 180/480 | klcA (chromosome) |
|  | A→G | 340/363 | hypothetical protein (chromosome) |
| CN31-pF65-tet(X4)-E100^+T-1^ | G→T | 102/135 | hypothetical protein |
|  | T→A | 21/261 | trbE |
|  | G→A | 15/282 | traQ |
|  | A→G  A→G | 369/777  399/777 | traT |
|  | T→C  G→A  C→T  A→C  T→G  T→C  C→T  T→A  A→G  A→T  T→C  T→C  A→G  T→C  T→C | 204/2190  221/2190  345/2190  516/2190  1113/2190  1527/2190  1627/2190  1677/2190  1690/2190  1695/2190  1752/2190  1758/2190  1827/2190  1918/2190  2157/2190 | traD |
|  | A→C  C→T  T→G  C→T  A→G  T→C  T→A  G→C  T→C  A→G  A→C  T→C  C→A  C→T  A→G  T→C  A→G  T→C  T→A | 174/1878  243/1878  375/1878  466/1878  478/1878  489/1878  720/1878  969/1878  1035/1878  1065/1878  1071/1878  1098/1878  1173/1878  1367/1878  1386/1878  1494/1878  1554/1878  1611/1878  1669/1878 | traI |
|  | A→C | 987/1293 | 3-oxo-tetronate kinase(chromosome) |
|  | G→A | 180/480 | klcA (chromosome) |
|  | G→T | 201/435 | marR(chromosome) |
|  | A→G | 340/363 | hypothetical protein (chromosome) |
| CN31-pF65-tet(X4)-E100^+T-4^ | T→G  A→C  T→C  C→G | 706/1215  482/1215  439/1215  425/1215 | floR |
|  |  |  |  |
|  |  |  |  |
|  |  |  |  |
|  | T→A | 21/261 | trbE |
|  | T→C  G→A  A→C  T→G  T→C  C→T  T→A  A→G  A→T  T→C  T→C  A→G  T→C  T→C | 204/2190  221/2190  516/2190  1113/2190  1527/2190  1627/2190  1677/2190  1690/2190  1695/2190  1752/2190  1758/2190  1827/2190  1918/2190  2157/2190 | traD |
|  | A→C  C→T  T→G  C→T  A→G  T→C  T→A  G→C  T→C  A→G  A→C  T→C  C→A  C→T  A→G  T→C  A→G  T→C  T→A | 174/1878  243/1878  375/1878  466/1878  478/1878  489/1878  720/1878  969/1878  1035/1878  1065/1878  1071/1878  1098/1878  1173/1878  1367/1878  1386/1878  1494/1878  1554/1878  1611/1878  1669/1878 | traI |
|  | C→T | 1058/1362 | hypothetical protein |
|  | C→T | 278/1413 | adeQ(chromosome) |
|  | G→A | 180/480 | klcA (chromosome) |
|  | G→T | 201/435 | marR(chromosome) |
|  | A→G | 340/363 | hypothetical protein (chromosome) |
| CN31-pF65-tet(X4)-E100^+T-5^ | T→G  A→C  T→C  C→G | 706/1215  482/1215  439/1215  425/1215 | floR |
|  | G→T | 102/135 | hypothetical protein |
|  | T→A | 21/261 | trbE |
|  | G→A | 15/282 | traQ |
|  | A→G  A→G | 369/777  399/777 | traT |
|  | C→T  A→C  T→C  C→T  T→A  A→G  A→T  T→C  T→C  A→G  T→C | 345/2190  516/2190  1527/2190  1627/2190  1677/2190  1690/2190  1695/2190  1752/2190  1758/2190  1827/2190  2157/2190 | traD |
|  | A→C  C→T  T→G  C→T  A→G  T→C  T→A  G→C  T→C  A→G  A→C  T→C  C→A  C→T  A→G  T→C  A→G  T→C  T→A | 174/1878  243/1878  375/1878  466/1878  478/1878  489/1878  720/1878  969/1878  1035/1878  1065/1878  1071/1878  1098/1878  1173/1878  1367/1878  1386/1878  1494/1878  1554/1878  1611/1878  1669/1878 | traI |
|  | C→T | 1058/1362 | hypothetical protein |
|  | G→A | 180/480 | klcA (chromosome) |
|  | G→T | 201/435 | marR(chromosome) |
|  | A→G | 340/363 | hypothetical protein (chromosome) |

**Table S3. Functional prediction of D67E mutation in *marR* gene.**

| Variant | PROVEAN score | Prediction^1^ |
| --- | --- | --- |
| D67E | -3.697 | Deleterious |

^1^ Variants with a score equal to or below -2.5 are considered “deleterious,”

Variants with a score above -2.5 are considered “neutral”.


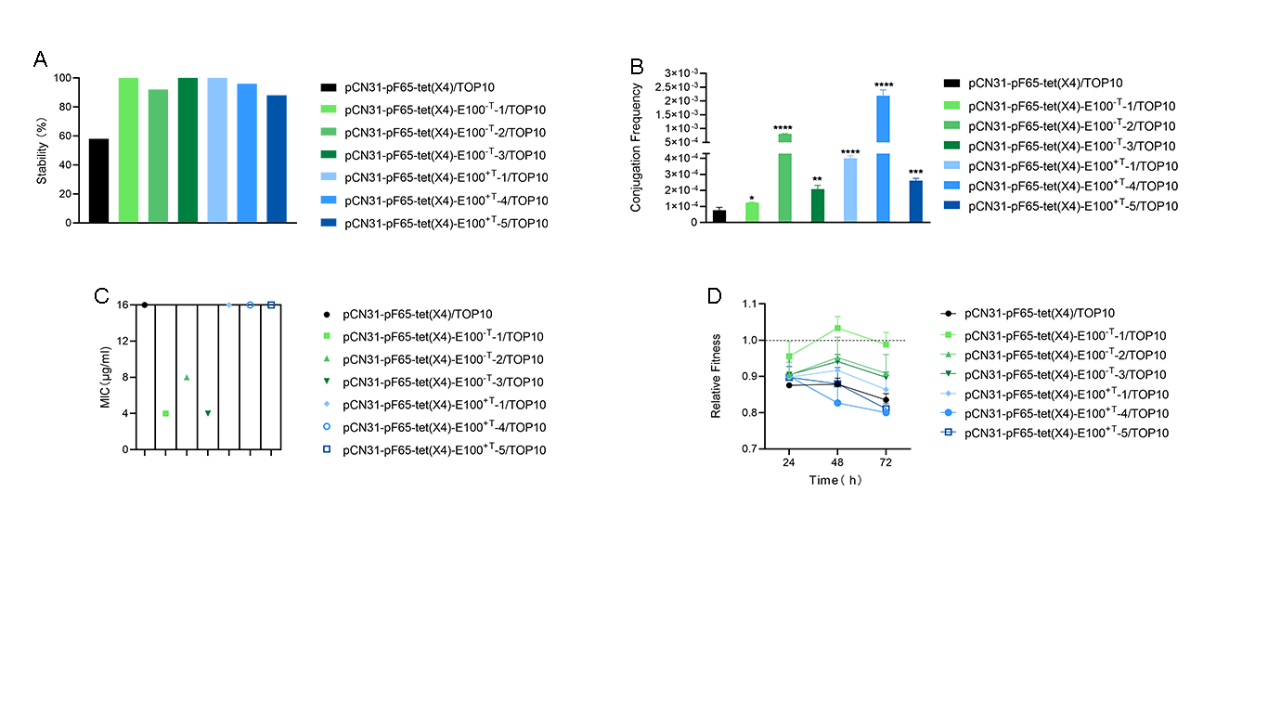
 **Figure S1 Phenotypic alteration of TOP10 after acquiring the evolved plasmids. (A)** Comparison of stability of the focal plasmid and *tet*(X4) gene between transformants carrying ancestral plasmid and carrying evolved plasmids. **(B)** Comparison of conjugation transfer between transformants carrying ancestral plasmid and carrying evolved plasmids.
